# Dietary baicalin supplementation increases the milk unsaturated fatty acid proportion through modulating milk metabolism and the microbiome in dairy cows

**DOI:** 10.1128/spectrum.00836-26

**Published:** 2026-10-07

**Authors:** Yanjun Shen, Dongwen Dai, Haoqi Han, Yangyi Hao, Zhanshan Yang, Shengli Li

**Affiliations:** 1 State Key Laboratory of Animal Nutrition and Feeding, Beijing Engineering Technology Research Center of Raw Milk Quality and Safety Control, College of Animal Science and Technology, China Agricultural University 462230, Beijing, China; 2 Qinghai Academy of Animal Husbandry and Veterinary Sciences, Qinghai University 207475, Xining, Qinghai, China; Nanjing Agricultural University, Nanjing, China

**Keywords:** baicalin, dairy cow, milk, fatty acid, milk microbiome

## Abstract

The composition and characteristics of milk fatty acids, which play a pivotal role in nutrition, are profoundly influenced by the dietary components consumed by cows. Like other flavonoids, baicalin (BAI) may affect the proportion of milk fatty acids. This study evaluates the effect of dietary supplementation with BAI on milk microbiota and host metabolism to modulate milk fatty acid composition. Results indicated that dietary supplementation with BAI increased proportions of unsaturated fatty acids (UFAs) (e.g., C18:1n9c and C18:2n6c). BAI reshaped the milk microbiome, wherein enriched taxa such as *Myroides* were positively associated with increased milk UFA and altered milk metabolite profiles. Additionally, BAI regulated some metabolites (e.g., alpha-KG) in milk, which were linked to promoting the synthesis of UFA, and regulated host metabolism (e.g., primary bile acid biosynthesis) that enhanced UFA metabolism. Together, these findings demonstrate the great potential of BAI in effectively modifying the milk fatty acid profile to enhance its nutritional quality.

**IMPORTANCE:**

Milk is a major source of dietary fat for humans, and increasing the proportion of unsaturated fatty acids (UFAs) in milk can improve its nutritional quality. This study demonstrates that dietary supplementation with baicalin, a natural flavonoid from Scutellaria baicalensis, effectively increases milk UFA content in dairy cows. The findings reveal that baicalin modulates milk fatty acid composition through reshaping the milk microbiome (e.g., enriching *Myroides*) and altering mammary and systemic metabolism. These results provide a novel, plant-based nutritional strategy for enhancing milk quality and offer mechanistic insights into the interplay between dietary bioactive compounds, mammary microbiota, and lipid metabolism in lactating dairy cows.

## INTRODUCTION

Milk and dairy products constitute significant dietary sources of protein, fat, minerals, vitamins A and B, and other indispensable nutrients for humans. They are not only nutritionally critical animal-based foods but also serve as a dietary cornerstone for people globally. With rising population growth and evolving nutritional priorities, the global milk demand is projected to increase (1). Therefore, strategies to enhance the quality of milk and dairy products have become a pivotal research focus in food science and agricultural technology. Oleic acid (OA) present in dairy milk demonstrates hypolipidemic effects, evidenced by its capacity to decrease low-density lipoprotein cholesterol (LDL-C) and triglyceride levels while elevating high-density lipoprotein cholesterol (HDL-C) concentration, consequently attenuating atherosclerosis development (2). The conjugated linoleic acid (CLA) in milk and dairy products can modulate inflammatory factors to alleviate chronic inflammation (3). Therefore, the lipid profile present in milk plays a crucial role in shaping the nutrition and sensory quality of milk and dairy products (4). Dietary components of cows significantly impact milk quality, and certain feed additives can effectively improve the fatty acid composition of milk, thereby providing a feasible strategy for modulating dairy cow production of high-quality milk (5).

Flavonoids, as natural bioactive compounds, have emerged as novel feed additives applicable in dairy cows, demonstrating potential to enhance milk quality (6), modulate rumen microbiota (7), improve antioxidant capacity and immune regulation, and benefit metabolism in dairy cows (8). Besides, flavonoids are highly degradable and do not accumulate through the food chain, ensuring their safety for milk production (9). Therefore, flavonoids are increasingly regarded as valuable dietary supplements for applications in milk production. For milk fatty acid composition, some studies indicated that flavonoids and feed rich in flavonoids may affect it. Zhang et al. (10) demonstrated that dietary supplementation with 8 g/kg of flavonoid-rich extracts (from propolis and red clover isoflavone) elevated the milk unsaturated fatty acid (UFA) content. The effects of flavonoid-rich feed (hybrid broussonetia papyrifera fermented feed) were also investigated by Zhao et al. (11), who recorded that UFA in milk was increased. The above results indicate that flavonoids may elevate the UFA content in milk. Multiple studies have demonstrated that fatty acids in milk are closely linked to host metabolism and microbial activity (12, 13). Besides, the advancement of microbiome and metabolomics technologies offers novel mechanistic insights into how flavonoid compounds influence fatty acid profiles in milk. Current findings in lactogenesis research elucidate that flavonoid-rich extracts from citrus peel and bamboo leaf can provide a valuable reference for understanding alterations in the milk microbiome and metabolome (1, 8). Therefore, flavonoids may modulate milk fatty acid composition by exerting effects on host metabolism and milk microbiota.

Baicalin (7-O-β-d-glucuronic acid, BAI), the principal flavonoid extracted from *Scutellaria baicalensis*, a traditional Chinese medicinal herb, possesses oxidation-resistant, inflammation-reducing, and antibacterial properties (14). In dairy production, *Scutellaria baicalensis* and its extracts have been shown to prevent and treat mastitis, reduce the impact of heat stress, and improve milk yield (15). Like other flavonoids, BAI may affect host metabolism and milk microbiota to modulate milk fatty acid composition. Therefore, this study was designed to assess the effects of BAI supplementation on milk microbes, host metabolome, and milk metabolome. We further hypothesized that cows supplemented with BAI could regulate milk fatty acid composition.

## MATERIALS AND METHODS

### Baicalin (BAI) preparation, animals, and experimental design

BAI was commercially sourced from Shanxi Snout Biotechnology Co., Ltd., located in Baoji, Shaanxi, China. The total flavonoids (85.13%) in the final product were quantified according to the methodology established by Pitz et al. (16). Table S1 lists the chemical constituents of BAI.

The experiment was performed from July to September 2023. Six Holstein cows (2.5 ± 0.5 parity, 680 ± 36 kg of BW, and 84 ± 7 days in milk) participated in a crossover design comprising 28-day cycles, with an initial 21 days for adaptation followed by 7 days dedicated to sample collection. Cows were allocated to two feeding regimens randomly: a basal diet for the CON group, and the basal diet plus 25 g/day of BAI at the individual level for the BAI group. The supplement was divided daily by top-dressing and mixed with a small amount of the feed portion provided in the morning. The cows were fed twice per day at 08:00 and 15:00, with feed supplied at 10% above the actual consumption level. Milking was performed twice daily, at 08:00 and 20:00, using manual portable milking equipment (SC23, Zibo Zhisong Livestock Equipment Co., Ltd., Zibo, Shandong, China), with yield data manually documented. The formulated diet contained complementary nutrients in compliance with NASEM (2021) standards (Table S2).

### Milk and blood sampling and analysis of milk fatty acid profiles

Blood and milk samples were recorded on day 26 of each period. To ensure the quality of milk samples for microbiome and metabolome analysis, pre-milking operations were performed according to the study of Ganda et al. (17). Blood samples were centrifuged (3,000 × *g*, 15 min, 4°C) to obtain serum; additionally, serum and milk samples were stored at −80°C. The fatty acid profiles of the milk samples were analyzed using a GC-MS system (Agilent 7890A/5975C, Agilent Technologies, Santa Clara, CA, USA) and expressed as FAME as per the manufacturer’s protocol (18).

### 16S rRNA gene sequencing and bioinformatic analysis of milk microbiota

The isolation of genomic DNA from milk was performed using the E.Z.N.A. Soil DNA Extraction Kit (Omega Bio-Tek, Norcross, GA, USA) in accordance with the guidelines of Yang et al. (19). Using the primer pair 338F (5′-ACTCCTACGGGAGGCAGCAG-3′) and 806R (5′-GGACTACHVGGGTWTCTAAT-3′), the V3–V4 regions of bacterial 16S rRNA genes were amplified in triplicate. The PCR amplification system was assembled following the protocol detailed by Kong et al. (20). Following electrophoresis using a 2% agarose gel, the amplicons underwent purification via the AxyPrep DNA extraction kit (Thermo Fisher Scientific, Waltham, MA, USA), and their concentrations were subsequently measured with QuantiFluor-ST (Promega Corporation, Madison, WI, USA). The Illumina MiSeq PE300 platform (Illumina Inc., San Diego, CA, USA) was employed to prepare paired-end sequencing libraries, with the technical service supported by Shanghai Biozeron Biotechnology Co., Ltd. The sequencing raw data are publicly accessible in the NCBI SRA database under accession code PRJNA1332947.

Paired-end reads were demultiplexed and then trimmed of barcode and primer sequences. Overlapping paired-end fragments were computationally reconstructed through FLASH software (version 1.2.11) before being analyzed in QIIME2 (version 2022.2) for sample-specific sequence segregation (21). The raw sequencing reads were processed with the DADA2 plugin in QIIME2 for sequence filtering, denoising, chimera detection, and amplicon sequence variant (ASV) generation (22). ASVs were taxonomically classified using the SILVA database (version 138), and microbial species composition across taxonomic ranks (phylum to species) was obtained (19). QIIME2 (version 2022.2) was also used for calculating α-diversity indices (Chao1, Simpson, and Shannon indices). Microbial β-diversity was evaluated using Bray-Curtis distances, with principal coordinate analysis (PCoA) and analysis of similarity (ANOSIM). Linear discriminant analysis effect size (LEfSe) was applied to identify discriminative taxa between the various treatment groups (23).

### Serum and milk metabolomics analysis

The milk and serum samples underwent controlled thawing (4°C) followed by supplementation with 300 μL methanol and 10 μL L-2-chlorophenylamine internal standard in a centrifuge tube containing precisely measured 100 mg aliquots. The mixture was vortexed for 60 s and centrifuged (12,000 × *g*, 10 min, 4°C) to collect the supernatant, which was then dried in a SpeedVac concentrator (H_2_O mode) for LC-MS analysis. The dried extract was rehydrated using an 80% methanol aqueous solution containing 2-chloro-L-phenylalanine, with subsequent sterile filtration through 0.22-μm pore-size membranes. Finally, 4.0 μL of the analyte solution was introduced into a Thermo Scientific UltiMate 3000 UHPLC system (Thermo Fisher Scientific), integrated with a TripleTOF 5600+ mass spectrometer (SCIEX, Framingham, MA, USA) via a DuoSpray ion source operating in electrospray ionization mode. Chromatographic separation was conducted employing a Waters ACQUITY BEH Amide analytical column (100 mm length × 4.6 mm internal diameter packed with 3 μm particles). The chromatographic conditions, including the mobile phase composition, flow rate, and gradient program, were implemented as specified by Dai et al. (24). The raw metabolomic data from serum and milk samples were collected and processed by Compound Discoverer software (Thermo Fisher Scientific). Metabolites were annotated by matching their exact mass-to-charge ratios (*m/z*), observed chromatographic retention times, and respective peak areas. The Kyoto Encyclopedia of Genes and Genomes (KEGG) and the Human Metabolome Database were employed to align with mass spectral data for metabolite identification.

Principal component analysis (PCA) was conducted via SIMCA-P software (version 11.0; Umetrics AB, Umeå, Sweden). Differentially expressed metabolites (DEMs) were detected according to a combination of variable importance in projection (VIP) >1.0, fold change (FC) >1.2 or FC <0.8, and *P* values (<0.01) confirmed by Student’s *t*-test. The Student’s *t*-test was applied to identify significant differential metabolites in serum and milk, followed by *P* value adjustment using the false-discovery-rate correction. Pathway enrichment investigations were conducted by the MetaboAnalyst 5.0 platform (https://www.metaboanalyst.ca) with the *Bos taurus* KEGG library to interrogate inter-group differences (25).

### Statistical analysis

The analysis of milk fatty acid proportions was conducted with R (version 4.4.3) using Student’s paired *t*-test to compare the differences between groups. The PCA of the milk fatty acid proportions was also performed via SIMCA-P software (version 11.0; Umetrics AB). To assess correlations among the milk and serum metabolomes, milk microbiome, and milk fatty acid profile, the Mantel and Spearman’s rank correlation tests were performed, followed by the generation of correlation heatmaps, using the online tool ChiPlot (https://www.chiplot.online/). Significant differences were defined as *P* < 0.05.

## RESULTS

### Effect of baicalin on milk fatty acid proportion

**Fig 1:**
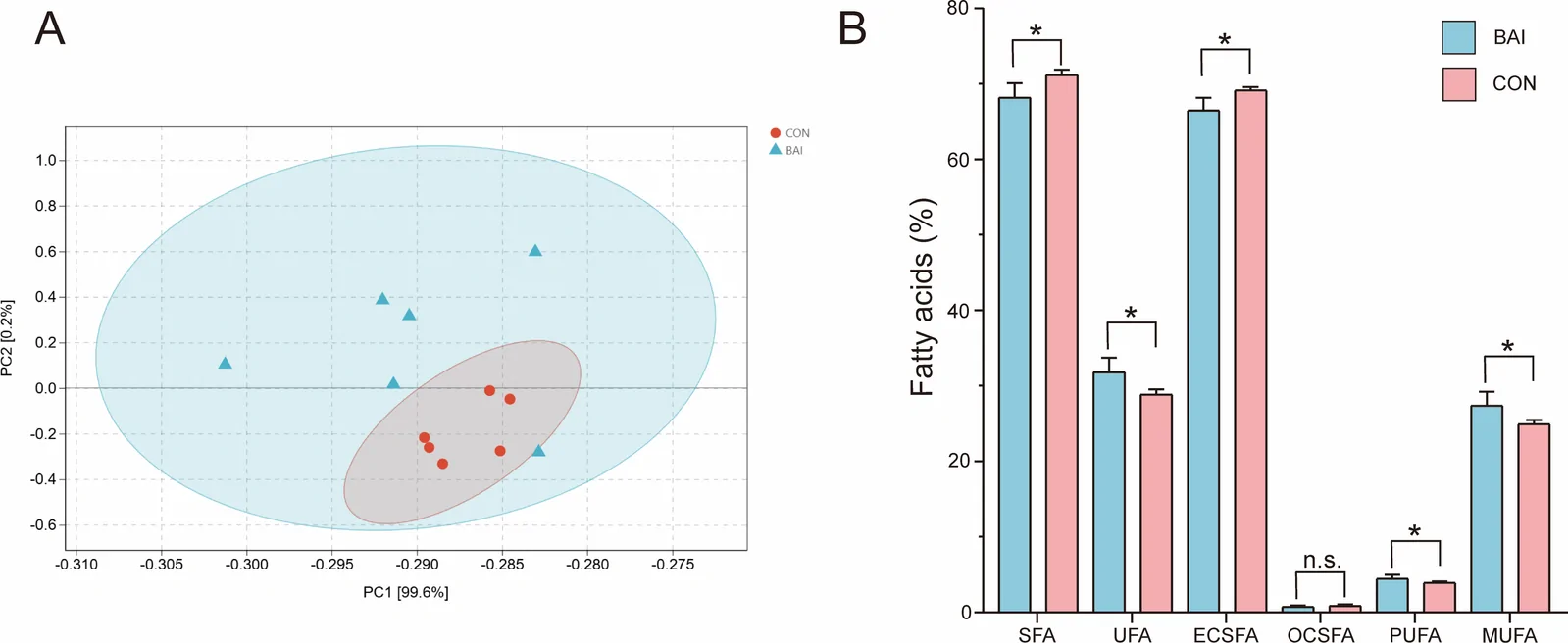
Effects of dietary baicalin supplementation on the milk fatty acid proportion in dairy cows. (**A**) Principal component analysis diagram of the fatty acid proportion in milk. (**B**) Effect of baicalin on proportions of saturated fatty acid (SFA), unsaturated fatty acid (UFA), even-chain saturated FA (ECSFA), odd-chain saturated FA (OCSFA), monounsaturated FA (MUFA), and polyunsaturated FA (PUFA) in milk. * indicates a significant difference between the CON and BAI groups (*P* < 0.05). n.s. indicates no significant difference (*P* > 0.05). CON = control diet with no baicalin (BAI) supplementation (*n* = 6); BAI = control diet with 25 g/day BAI supplementation per cow (*n* = 6).

Fatty acids were identified and analyzed by PCA to identify potential relationships between the supplementation of baicalin and bovine milk fatty acids. Analysis revealed no significant variation in the two principal components (Fig. 1A). The effects of feeding BAI on the milk fatty acid proportion of dairy cows were presented in Table 1. Among all fatty acids, C16:0 was the most prevalent fatty acid. Compared with the CON group, BAI supplementation significantly increased proportions of C18:1n9c, C18:2n6c, and C18:3n3 but lowered proportions of C12:0 and C18:0 (*P* < 0.05). Other significant changes in total proportions of fatty acids were observed following baicalin supplementation in the cows' diet, characterized by a significant increase in the total proportions of UFA and a substantial decrease in those of saturated fatty acid (SFA) (*P* < 0.01) (Fig. 1B). In addition, the BAI group exhibited a significantly lower concentration of even-chain saturated fatty acid (ECSFA) (*P* < 0.01) along with higher proportions of both monounsaturated fatty acid (MUFA) and polyunsaturated fatty acid (PUFA) (*P* < 0.05) relative to the CON group, whereas the total proportion of odd-chain saturated fatty acid (OCSFA) was not influenced by baicalin (Fig. 1B).

**TABLE 1:** Effect of feeding baicalin on the milk fatty acid proportion of dairy cows^*b*^

Fatty acids (%)	Treatment groups^*a*^		SEM	*P* value
	BAI	CON		
C6:0	1.48	1.50	0.778	0.778
C8:0	1.09	1.14	0.485	0.485
C10:0	2.76	2.99	0.146	0.146
C12:0	3.41	3.84	0.011	0.011
C13:0	0.13	0.16	0.248	0.248
C14:0	11.45	11.28	0.626	0.626
C14:1	0.84	0.86	0.866	0.866
C15:0	0.95	1.18	0.175	0.175
C16:0	36.48	37.16	0.379	0.379
C16:1	1.69	1.59	0.328	0.328
C17:0	0.44	0.52	0.125	0.125
C18:0	9.49	10.90	0.035	0.035
C18:1n9c	24.80	22.41	0.018	0.018
C18:2n6c	3.79	3.29	0.035	0.035
C18:3n3	0.29	0.24	0.014	0.014
C20:0	0.15	0.17	0.166	0.166
C20:1	0.05	0.07	0.218	0.218
C20:3n6	0.01	0.04	0.312	0.312
C20:4n6	0.16	0.14	0.312	0.312
C21:0	0.21	0.23	0.144	0.144
C22:0	0.12	0.07	0.324	0.324
C23:0	0.15	0.14	0.721	0.721
C24:0	0.06	0.08	0.170	0.170

^*a*^ CON = control diet with no baicalin (BAI) supplementation (*n* = 6); BAI = control diet with 25 g/day BAI supplementation per cow (*n* = 6).

^*b*^ The group of the same type of fatty acids with different letters was significantly different (*P* < 0.05).

### Taxonomic configurations of milk bacteria

**Fig 2:**
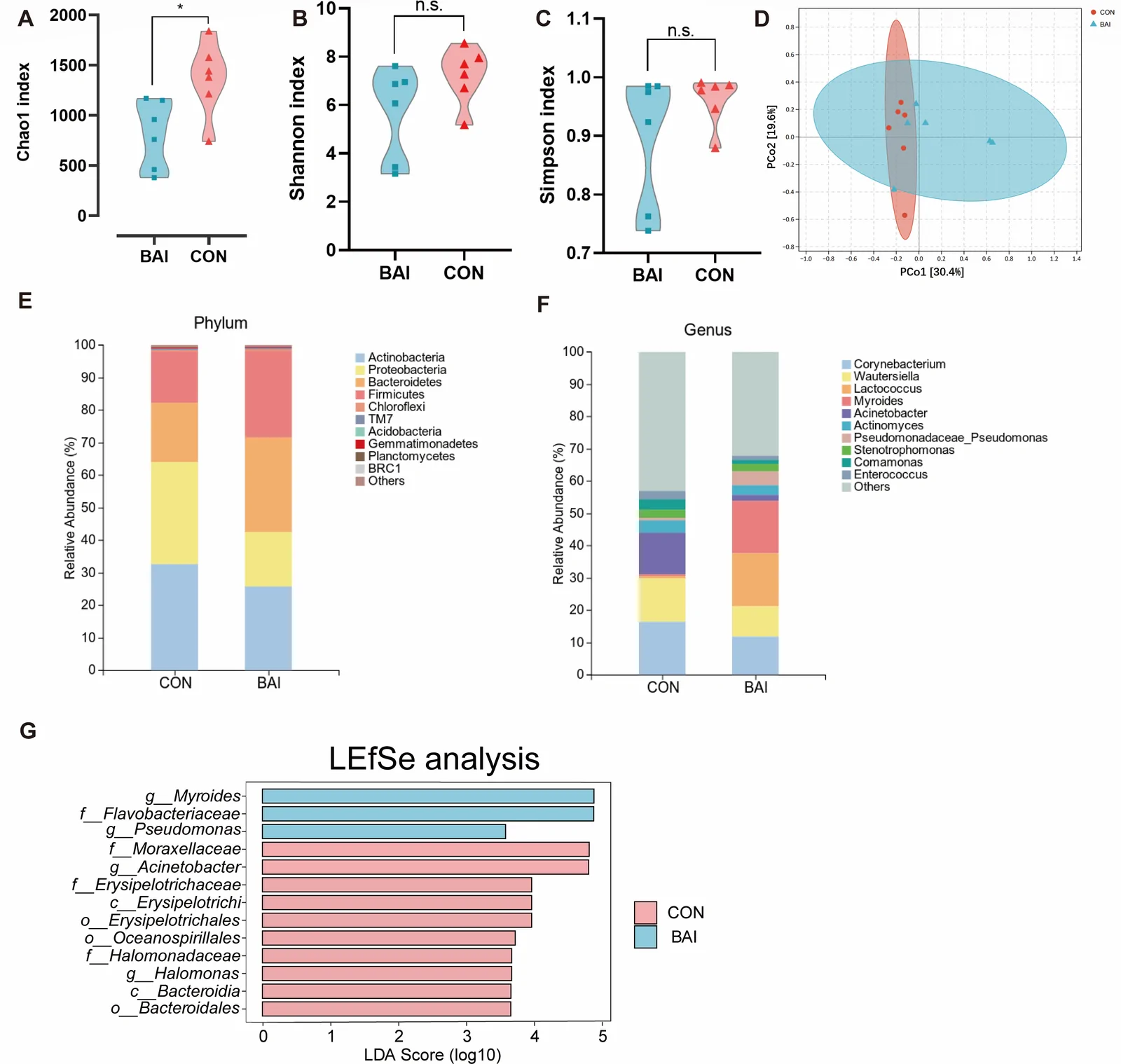
Effects of dietary baicalin supplementation on the diversity and composition of the milk bacterial community in dairy cows. (**A**) Chao1 indices; (**B**) Shannon indices; (**C**) Simpson indices; (**D**) beta diversity based on principal coordinate analysis (PCoA) using weighted bray-Curtis; (**E**) relative abundance of bacteria community at the phylum level; (**F**) relative abundance of bacteria community at the genus level; and (**G**) linear discriminant analysis effect size (LEfSe) approach identifying biomarker genera between two groups. CON = control diet with no baicalin (BAI) supplementation (*n* = 6); BAI = control diet with 25 g/day BAI supplementation per cow (*n* = 6).

The effect of BAI addition to the diet of cows on milk microbes was assessed via 16S rRNA high-throughput sequencing. The Chao1 index significantly differed between the CON and BAI groups, but the Shannon and Simpson indices did not (Fig. 2A through C). The β-diversity of milk bacteria was analyzed by PCoA, explaining 19.6% and 30.4% of the variance, respectively, but no clear clustering between the CON and BAI groups was detected (ANOSIM: *P*  =  0.096) (Fig. 2D). *Firmicutes*, *Bacteroidetes*, *Actinobacteria*, and *Proteobacteria* were the dominant bacterial phyla in the two groups (Fig. 2E). The dominant bacterial genera identified across both groups included *Corynebacterium*, *Wautersiella*, *Lactococcus*, *Myroides*, *Acinetobacter*, and *Actinomyces* (Fig. 2F). We used LEfSe analysis (LDA > 3.5, *P* < 0.05) to discern discriminative bacterial taxa between the CON and BAI groups, which identified 13 microbial biomarkers, as visualized in Fig. 2G. At the family level, *Flavobacteriaceae* was enriched in the BAI group; meanwhile, *Moraxellaceae*, *Erysipelotrichaceae*, and *Halomonadaceae* were enriched in the CON group. At the genus level, *Myroides* and *Pseudomonas* were enriched in the BAI group; meanwhile, *Acinetobacter* and *Halomonas* were enriched in the CON group.

### Milk metabolomics analysis

**Fig 3:**
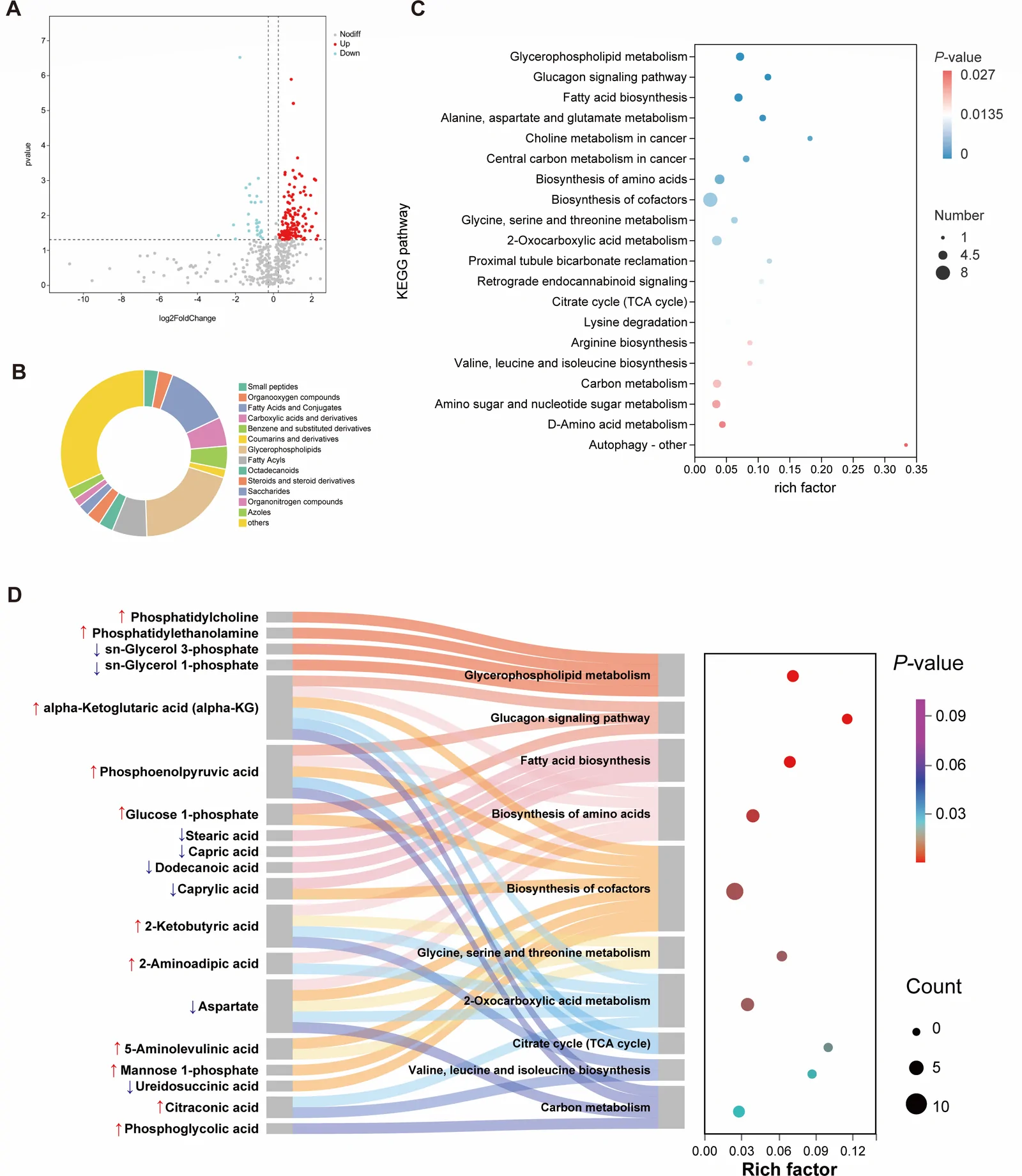
Effect of dietary baicalin supplementation on milk metabolomics in dairy cows. (**A**) Volcano map of differentially expressed metabolites (DEMs) between the treatment groups of different milks. Upregulated metabolites are indicated by red dots, downregulated ones are indicated by blue dots, and those that are not significantly different are indicated by gray dots. (**B**) Pie chart classifying the 177 identified differential metabolites. (**C**) KEGG enrichment pathway analysis. KEGG, Kyoto Encyclopedia of Genes and Genomes. (**D**) The 19 differential milk metabolites are categorized into different fatty acid metabolism-related pathways [based on KEGG enrichment pathway analysis (**C**)]. Red upward arrows indicate metabolite upregulation, and blue downward arrows represent metabolite downregulation. CON = control diet with no baicalin (BAI) supplementation (*n* = 6); BAI = control diet with 25 g/day BAI supplementation per cow (*n* = 6).

The unsupervised PCA plots failed to clearly distinguish the CON and BAI milk metabolome in positive and negative ion modes (Fig. S1C and D). A total of 177 DEMs were identified under the criteria of VIP > 1, FC > 1.2, or FC < 0.8, and corrected *P* value < 0.05 (Fig. 3A; Table S4), and these metabolites were subsequently divided into 14 different classes (Fig. 3B). Metabolic pathway analysis based on the 177 DEMs revealed the enrichment of the pathways, and the top 20 pathways were all significantly enriched (*P* < 0.05) (Fig. 3C). Among them, 10 fatty acid metabolism-related pathways, including glycerophospholipid metabolism, glucagon signaling pathway, fatty acid biosynthesis, biosynthesis of amino acids, biosynthesis of cofactors, glycine, serine, and threonine metabolism, 2-oxocarboxylic acid metabolism, citrate cycle (TCA cycle), valine, leucine, and isoleucine biosynthesis, and carbon metabolism, were extremely significantly enriched (*P* < 0.01) (Fig. 3D). In the 19 metabolites involved in these pathways, concentrations of phosphatidylcholine, phosphatidylethanolamine, alpha-ketoglutaric acid (alpha-KG), phosphoenolpyruvic acid, glucose 1-phosphate, 2-ketobutyric acid, 2-aminoadipic acid, 5-aminolevulinic acid, mannose 1-phosphate, citraconic acid, and phosphoglycolic acid were higher in the BAI group (*P* < 0.05), while sn-glycerol 3-phosphate (sn-G3P), sn-glycerol 1-phosphate (sn-G1P), stearic acid, capric acid, dodecanoic acid, caprylic acid, aspartate, and ureidosuccinic acid were significantly reduced (*P* < 0.01) (Fig. 3D).

### Serum metabolomics profiling

**Fig 4:**
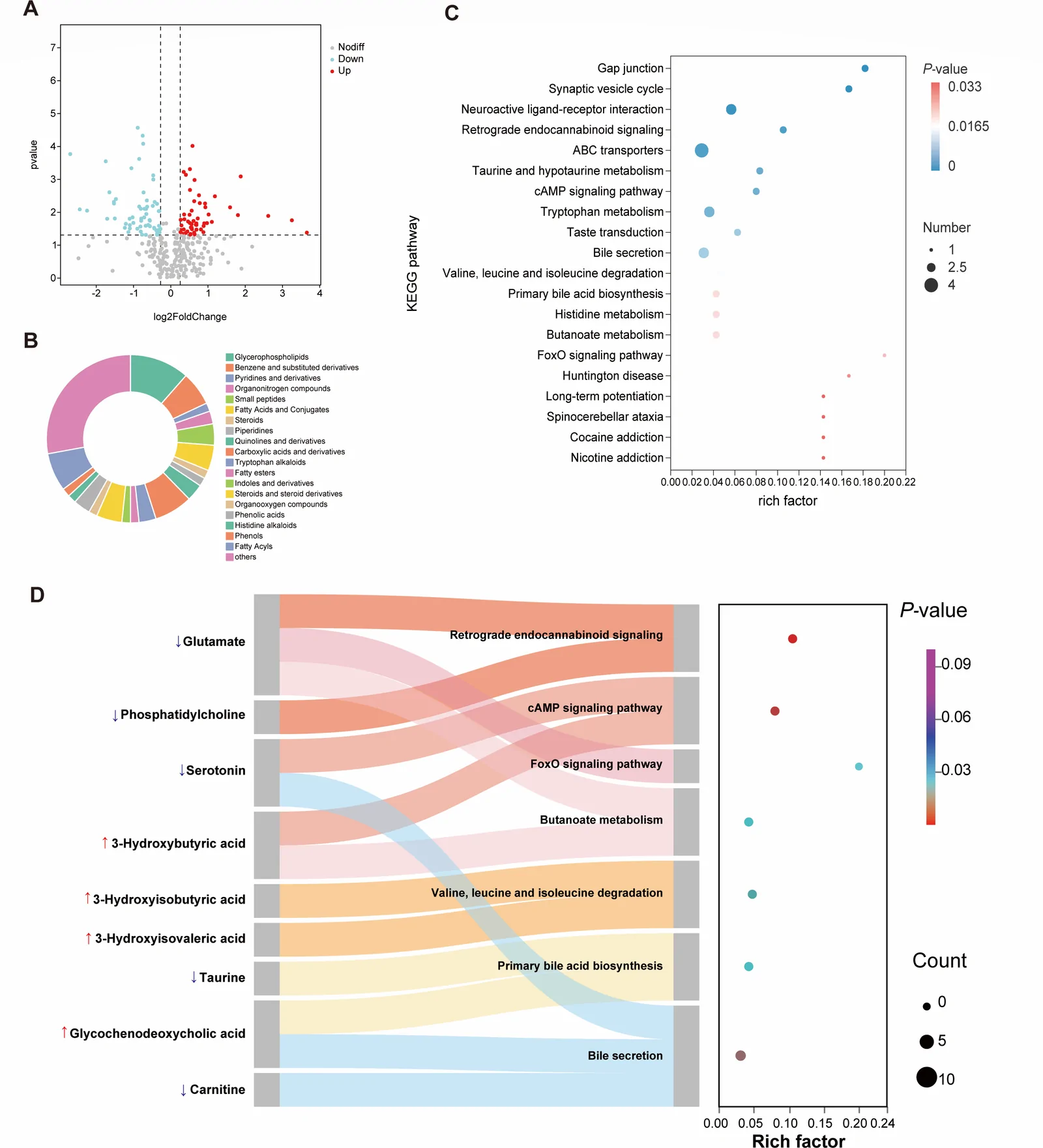
Effect of dietary baicalin supplementation on serum metabolomics in dairy cows. (**A**) Volcano map of differentially expressed metabolites (DEMs) between treatment groups of different serums. Upregulated metabolites are indicated by red dots, downregulated ones are indicated by blue dots, and those that are not significantly different are indicated by gray dots. (**B**) Pie chart classifying the 122 identified differential metabolites. (**C**) KEGG enrichment pathway analysis. KEGG, Kyoto Encyclopedia of Genes and Genomes. (**D**) The nine differential serum metabolites are categorized into different fatty acid metabolism-related pathways [based on KEGG enrichment pathway analysis (**C**)]. Red upward arrows indicate metabolite upregulation, and blue downward arrows represent metabolite downregulation. CON = control diet with no baicalin (BAI) supplementation (*n* = 6); BAI = control diet with 25 g/day BAI supplementation per cow (*n* = 6).

The unsupervised PCA demonstrated distinct clustering of the serum metabolomes from the CON and BAI groups, both in the positive and negative ion modes (Fig. S1A and B). A total of 122 DEMs were identified under the criteria of VIP > 1, FC > 1.2, or FC < 0.8, and corrected *P* value < 0.05 (Fig. 4A; Table S3), and these metabolites were subsequently divided into 14 different classes (Fig. 4B). Metabolic pathway analysis based on the 122 DEMs revealed the enrichment of the pathways, and the top 20 pathways were all significantly enriched (*P* < 0.05) (Fig. 4C). Among them, seven fatty acid metabolism-related pathways, including retrograde endocannabinoid signaling, cAMP signaling pathway, FoxO signaling pathway, butanoate metabolism, valine, leucine, and isoleucine degradation, primary bile acid biosynthesis, and bile secretion, were extremely significantly enriched (*P* < 0.01) (Fig. 4D). In the nine metabolites involved in these pathways, concentrations of 3-hydroxybutyric acid, 3-hydroxyisobutyric acid, 3-hydroxyisovaleric acid, and glycochenodeoxycholic acid were higher in the BAI group (*P* < 0.05), while glutamate, phosphatidylcholine, serotonin, taurine, and carnitine were significantly reduced (*P* < 0.01) (Fig. 4D).

### Correlation analysis amongst milk and serum metabolome, milk microbiome, and milk fatty ratio

**Fig 5:**
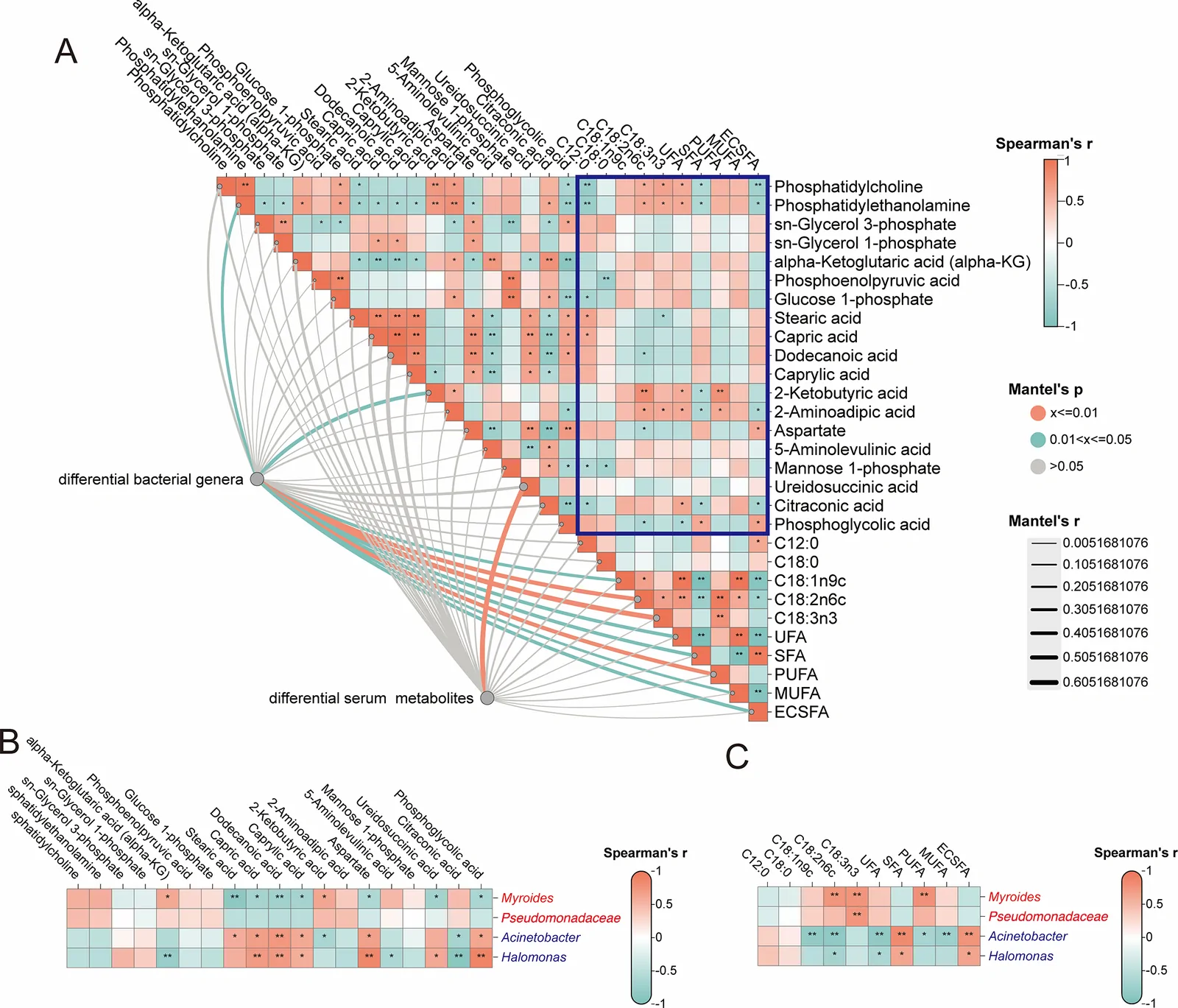
Correlation analysis amongst milk metabolome, serum metabolome, milk microbiome, and milk fatty ratio. (**A**) Spearman correlation analysis between 19 differential milk metabolites involved in fatty acid metabolism-related pathways and milk fatty ratio in the blue rank. Differential bacterial genera in milk [based on linear discriminant analysis effect size approach (Fig. 2G)] and the seven differential serum metabolites (Fig. 4D) were related to the 19 differential milk metabolites (Fig. 3D) and milk fatty ratio (Fig. 1B; Table 1) by partial (geographic distance corrected) Mantel tests. Edge width corresponds to Mantel’s *r* statistic for the corresponding distance correlations, and edge color denotes the statistical significance based on 9,999 permutations. (**B**) Spearman’s correlation heatmaps of the 19 differential milk metabolites and differential bacterial genera in milk. Red text indicates bacterial genera enriched in the BAI group, while blue text denotes those enriched in the CON group. (**C**) Spearman’s correlation heatmaps of milk fatty acid ratio and differential bacterial genera in milk. The red and blue text used here are consistent with those in panel **B**. CON = control diet with no baicalin (BAI) supplementation (*n* = 6); BAI = control diet with 25 g/day BAI supplementation per cow (*n* = 6). *, *P* < 0.05; **, *P* < 0.01 (Spearman's correlation).

Spearman correlation analysis was applied to investigate associations between milk metabolites and milk fatty acid proportion (blue rank in Fig. 5A). Nineteen metabolites were selected in the milk metabolome involved in fatty acid metabolism-related pathways (Fig. 3D). Fatty acid proportions and proportions that showed significant differences were selected (Fig. 1B; Table 1). To evaluate associations, we performed Mantel tests between distance matrices of serum metabolites and milk bacterial genera against those of differential milk metabolites and milk fatty acid proportions (Fig. 5A). At the genus level, *Myroides*, *Pseudomonas*, *Acinetobacter*, and *Halomonas* were selected in the milk microbiome as differential bacterial genera (Fig. 2G). Nine metabolites were selected in the differential serum metabolome, which were involved in fatty acid metabolism-related pathways (Fig. 4D).

As shown in Fig. 5A, the differential bacterial genera were correlated with the milk fatty acid proportion of C18:1n9c, C18:2n6c, C18:3n3, UFA, SFA, PUFA, MUFA, and ECSFA (Fig. 5A). Besides, for the two differential metabolites correlated with differential bacterial genera, there were correlations with the milk fatty acid proportion (Fig. 5A). Phosphatidylethanolamine was negatively correlated with the milk fatty acid proportion of C12:0, SFA, and ECSFA, and was positively correlated with the milk fatty acid proportion of C18:2n6c, C18:3n3, and UFA (*P* < 0.05). 2-Ketobutyric acid was negatively correlated with the milk fatty acid proportion of SFA and positively correlated with C18:2n6c, UFA, and PUFA (*P* < 0.05). In contrast, the serum differential metabolites were only correlated with ureidosuccinic acid in the milk differential metabolites (*P* < 0.05); moreover, ureidosuccinic acid was not correlated with any milk fatty acid proportion (Fig. 5A). These results suggest that the effects of dietary baicalin on mammary gland metabolism and milk fatty acid profiles are more strongly mediated by the milk microbiota than by host metabolism itself. We therefore performed Spearman’s test-based correlation analyses between four differential bacterial genera and the altered metabolites in milk (Fig. 5B), with milk fatty acid proportion parameters (Fig. 5C).

As shown in Fig. 5B, *Myroides* was positively correlated with alpha-KG and 2-ketobutyric acid, and negatively correlated with stearic acid, capric acid, dodecanoic acid, caprylic acid, aspartate, ureidosuccinic acid, and phosphoglycolic acid (*P* < 0.05). *Acinetobacter* was negatively correlated with 2-ketobutyric acid and citraconic acid, and positively correlated with stearic acid, capric acid, dodecanoic acid, caprylic acid, aspartate, and phosphoglycolic acid (*P* < 0.05). *Halomonas* was negatively correlated with alpha-KG, 5-aminolevulinic acid, and citraconic acid, and positively correlated with capric acid, dodecanoic acid, caprylic acid, aspartate, ureidosuccinic acid, and phosphoglycolic acid (*P* < 0.05). As shown in Fig. 5C, *Myroides* was positively correlated with the milk fatty acid proportion of C18:2n6c, C18:3n3, and PUFA (*P* < 0.05). *Pseudomonas* was positively correlated with the milk fatty acid proportion of C18:3n3 (*P* < 0.05). *Acinetobacter* was negatively correlated with C18:1n9c, C18:2n6c, UFA, PUFA, and MUFA, and positively correlated with SFA and ECSFA (*P* < 0.05). *Halomonas* was negatively correlated with C18:2n6c and UFA, and positively correlated with SFA and ECSFA (*P* < 0.05). The results above indicated that baicalin supplementation in cows’ diet can alter the microbial composition of the mammary gland; furthermore, changes in the mammary microbiota may affect the ratio of milk fatty acids directly or by influencing mammary metabolism.

Meanwhile, some differential milk metabolites that were not correlated with the differential bacterial genera also exhibited a correlation with the ratio of milk fatty acids (Fig. 5A). Phosphatidylcholine was negatively correlated with the milk fatty acid proportion of C12:0, SFA, and ECSFA, and positively correlated with C18:2n6c, C18:3n3, and UFA (*P* < 0.05). Phosphoenolpyruvic acid was negatively correlated with the milk fatty acid proportion of C18:0 (*P* < 0.05). Glucose 1-phosphate was negatively correlated with the milk fatty acid proportion of C12:0 (*P* < 0.05). Results above may show that baicalin supplementation in cows’ diets can affect the ratio of milk fatty acids by influencing mammary metabolism directly.

## DISCUSSION

Although many flavonoid-rich extracts have been regarded as potential plant-derived feed supplements for enhancing the productivity and well-being of ruminants (26), limited research has examined the effects of baicalin on the fatty acid proportion of milk. Fatty acids are essential nutrients in the human diet, accounting for 45–55% of the total energy provided by milk (10). Higher dietary intake of SFA can cause a rise in serum total cholesterol, thereby increasing the risk of cardiovascular disease (27). In comparison, higher consumption of UFA can be tied to a diminished disease risk, notably cardiovascular disease and coronary heart disease (28). Dietary supplementation with 25 g/day of baicalin reduced the SFA content and concurrently increased the level of UFA in bovine milk in our study. This finding is consistent with Zhang et al. (29), who reported that dairy cows receiving propolis extracts rich in flavonoids (specifically red clover isoflavones) at rates of 8 g/kg of ingested dry matter influenced the milk fatty acid profile. We also found that the cows fed baicalin had higher OA, linoleic acid, alpha-linolenic acid, PUFA, and MUFA proportions in milk, which have been reported to be beneficial for human health (30, 31). In addition, recent findings emphasize that omega-9 monounsaturated fatty acids, such as OA, positively affect health functions as well as active intermediates generated during metabolic processes (32).

For the metabolites and lipids present in milk, there may be several significant sources: *de novo* mammary synthesis, microbial production in the lacteal lumen, dietary consumption, systemic circulation from extra-mammary tissue, and transported from the bloodstream (1, 33). Therefore, milk microbiota, serum metabolites, milk metabolites, and milk fatty acids may have some intricate connections.

The milk microbiota composition has been demonstrated to not only critically influence mammary health in lactating dairy cows but also modulate lipid metabolism within the mammary gland (11). In the present study, the bacterial community richness (Chao indices) was significantly lower in the BAI group. Similarly, ACE and Chao indices were lower when artemisinin (other plant extracts) was supplemented in the cows’ diet in the study by Hou et al. (34)*,* and milk SCC was also lower, which suggests that milk bacterial community richness is associated with milk SCC and mammary health. At the genus level, we used the LefSe analysis to identify biomarkers. *Myroides* and *Pseudomonas* were enriched in the BAI group; meanwhile, *Acinetobacter* and *Halomonas* were enriched in the CON group. *Myroides* has been shown to degrade carbohydrates, synthesize fatty acids, and further convert short-chain fatty acids into aromatic compounds (35, 36). In the present study, *Myroides* was significantly positively correlated with the proportion of C18:2n6c, C18:3n3, and PUFA in milk. Therefore, these correlations suggest that *Myroides* may affect the fatty acid proportion of milk. *Pseudomonas*, present at low levels, is detectable in the mammary microbiota of healthy dairy cows and can alter milk fatty acid profiles by producing lipolytic enzymes (37, 38). Based on our results, *Pseudomonas* was also significantly positively correlated with the proportion of C18:3n3 in milk. *Acinetobacter* has been detected in healthy mammary glands, but its abundance may increase significantly when cows develop mastitis (39). Furthermore, *Acinetobacter* may affect fatty acid metabolic pathways and trigger oxidative stress in the mammary gland of cows (12, 40). In the correlation analysis, *Acinetobacter* was significantly negatively correlated with the proportion of UFA, PUFA, and MUFA in milk. Thus, these correlations suggest that *Acinetobacter* might reduce UFA in milk. Previous studies have shown a causal link between *Halomonas* and cheese spoilage (41), and *Halomonas* was also significantly negatively correlated with the proportion of UFA in our study. Therefore, based on our correlation analysis, we hypothesize that dietary supplementation with baicalin may improve the quality of processed dairy products by reducing the abundance of *Halomonas*. Collectively, baicalin may affect milk fatty acid proportions by altering the milk microbiota.

In milk metabolomic data, alpha-KG, which is a metabolite significantly upregulated in the BAI group, can activate stearoyl-CoA desaturase 1, converting stearic acid (C18:0) to OA (C18:1), thereby promoting the synthesis of monounsaturated fatty acids (42). Additionally, its antioxidant properties help prevent the oxidation of unsaturated fatty acids (43). An earlier study also showed that the level of alpha-KG in milk was positively associated with the short and medium-chain fatty acids in milk fat (44). In the correlation analysis, alpha-KG was found to be significantly positively correlated with *Myroides* and negatively correlated with *Halomonas*. Moreover, the increased levels of alpha-KG and phosphoenolpyruvic acid (PEP) in the BAI group may enhance the TCA cycle by promoting the activity of citrate synthase to strengthen the coupling between the TCA cycle and fatty acid biosynthesis (45). In the correlation analysis, PEP was significantly negatively correlated with the proportion of C18:0 in milk. Therefore, the increased alpha-KG and PEP in milk suggested that baicalin enhanced dairy cows' synthesis and metabolism of UFA by affecting milk microbiota composition. Meanwhile, we observed that baicalin may enhance the biosynthesis of amino acids (e.g, branched-chain amino acids), as evidenced by upregulated levels of alpha-KG, phosphoenolpyruvic acid, 2-ketobutyric acid, and 2-aminoadipic acid , and a downregulated level of aspartate. Recently, Xu et al. (46) found that branched-chain amino acids could alter acyl-carnitine, suggesting mammary fatty acid metabolism. In the correlation analysis, 2-ketobutyric acid, 2-aminoadipic acid, and citraconic acid were found to be significantly positively correlated with the proportion of UFA, and aspartate was negatively correlated with the proportion of C18:2n6c in milk. Furthermore, 2-ketobutyric acid was positively correlated with *Myroides* and negatively correlated with *Acinetobacter*. Conversely, aspartate was positively correlated with *Acinetobacter* and negatively correlated with *Myroides*. Based on our correlation analysis, we hypothesize that baicalin may affect fatty acid metabolism through amino acid metabolism in the mammary gland of dairy cows, and may be linked to milk microbiota composition.

We also detected that BAI could modulate glycerophospholipid metabolism by upregulating the content of PC and PE and downregulating sn-G3P and sn-G1P in milk metabolomic data. The biosynthesis of PC and PE competitively consumes sn-G3P, a critical precursor for glycerophospholipid synthesis (47). Concurrently, PC may suppress fatty acid catabolism by modulating the activity of carnitine palmitoyltransferase-1 (47), which may support a link with more UFAs in milk lipids. In the correlation analysis, PC and PE were also found to be significantly positively correlated with the proportion of C18:2n6c, C18:3n3, and UFA in milk. Therefore, the increased PC and PE in milk suggested baicalin enhancement on dairy cows' synthesis and metabolism of UFA. Meanwhile, the pathway of “Biosynthesis of cofactors” was significantly enriched based on the DEM between CON and BAI. The increase in alpha-KG, phosphoenolpyruvic acid, glucose 1-phosphate, and 5-aminolevulinic acid may enhance the biosynthesis of NAD^+^, FAD, and heme through this pathway (48–50). NAD^+^ promotes the activity of fatty acid desaturases, thereby improving the synthesis of monounsaturated fatty acids such as oleic acid (C18:1) (51). Concurrently, heme augments the catalytic function of cytochrome P450 enzymes, which mediate hydroxylation reactions of polyunsaturated fatty acids, including linoleic acid (C18:2) and α-linolenic acid (C18:3) (52). Therefore, baicalin may affect fatty acid metabolism by modulating cofactor biosynthesis in the bovine mammary gland. Besides, the decrease in stearic acid, capric acid, dodecanoic acid, and caprylic acid in the BAI group corresponded to the milk fatty ratio, reflecting the decline in milk SFA in the BAI group.

For serum metabolomic data, glutamate was a metabolite significantly downregulated in the BAI group. Its downregulation enhances lipid catabolism by attenuating retrograde endocannabinoid signaling and activating the FoxO signaling pathway (53, 54). 3-hydroxybutyric acid was increased in the BAI group, activating the peroxisome proliferator-activated receptor alpha signaling pathway to promote fatty acid oxidation (55). Meanwhile, we observed that baicalin could enhance valine, leucine, and isoleucine degradation, with the levels of 3-hydroxyisobutyric acid and 3-hydroxyisovaleric acid upregulated. Similarly, Zhao et al. (56) observed that citrus flavonoids promote branched-chain amino acid metabolism in dairy cows. Research has demonstrated that the metabolism of branched-chain amino acids may be associated with fatty acid metabolism (57, 58). Therefore, baicalin may enhance fatty acid metabolism through branched-chain amino acid metabolism in dairy cows. In our study, the pathways of “Primary bile acid biosynthesis” and “Bile secretion” were significantly enriched based on the DEM in serum between CON and BAI. Bile (including bile acid) is essential for lipid digestion and absorption in the intestine (59). Glycochenodeoxycholic acid is one of the bile acids that was upregulated in those two pathways in the BAI group, which could promote fatty acid oxidation by activating the farnesoid X receptor (FXR) and G protein-coupled bile acid receptor 1 (60). Consistently, Yang et al. (61) found that baicalin could indirectly regulate glycochenodeoxycholic acid via FXR. Therefore, baicalin may enhance fatty acid metabolism through bile acid metabolism in dairy cows.

**Fig 6:**
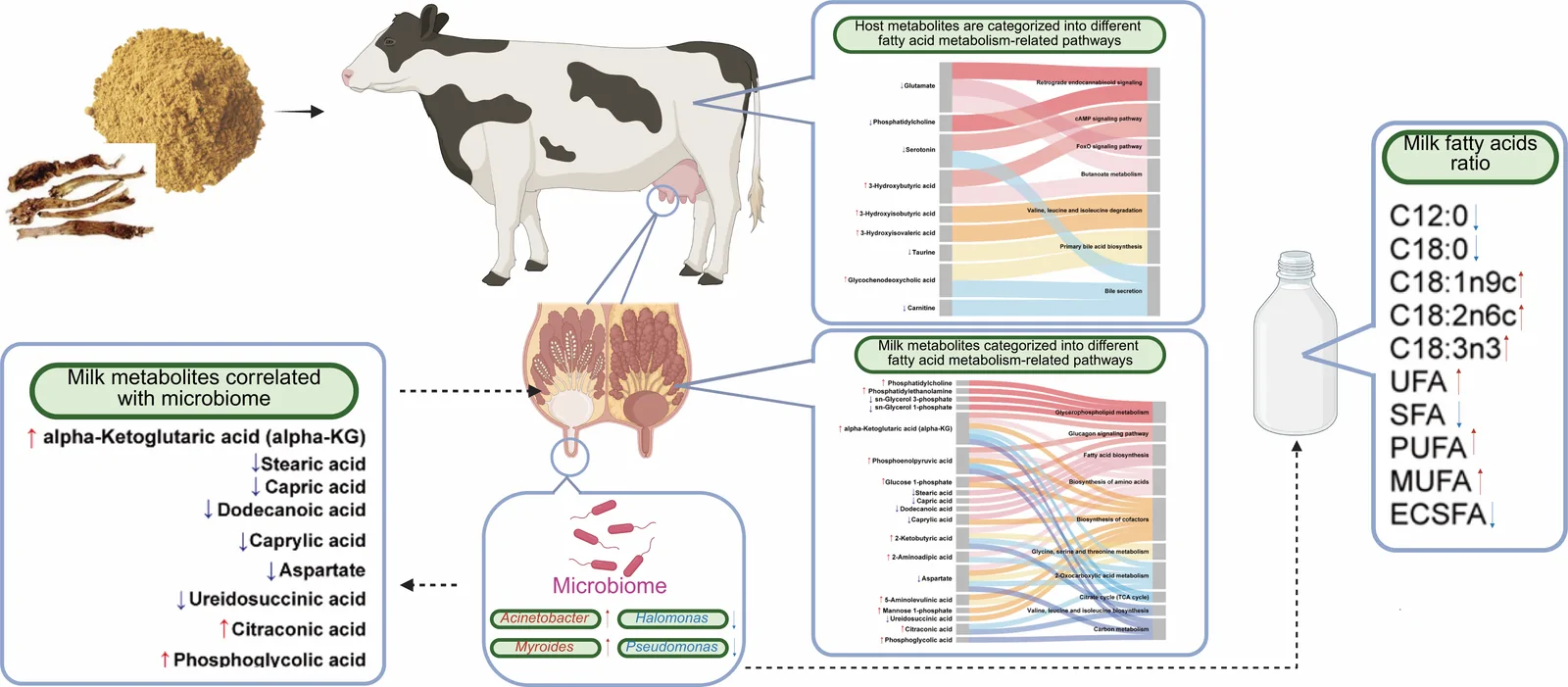
A summary of the results in the present study (created by BioRender.com). Upward (downward) solid arrows represent upregulated (downregulated) metabolites, microbes, and components in dairy cows and milk.

Combined, supplementing BAI in the diet may represent a viable strategy for increasing the proportion of milk UFA in lactating dairy cows. We suggest that the significant changes in milk microbiota, milk metabolites, and serum metabolites could be linked to milk fatty acid proportions (Fig. 6). In addition to the above-discussed observations, several limitations remain in this study. Rumen fermentation parameters and rumen microbiota were not characterized in this study. Given that ruminal biohydrogenation is a major determinant of milk fatty acid composition, the absence of these data limits our ability to fully discriminate between direct mammary effects and indirect rumen-mediated effects of baicalin. The milk microbiota may be influenced not only by dietary baicalin but also by external environmental factors (e.g., milking equipment, air, and teat skin), which were not controlled or monitored herein. The 16S rRNA sequencing cannot identify microbial metabolic pathways and accurately predict the metabolic pathways of different bacterial genera. Therefore, future investigations should simultaneously characterize rumen fermentation and microbiota to clarify the relative contributions of ruminal biohydrogenation and mammary metabolism to the altered milk fatty acid profile. In addition, strict control of environmental microbial sources and employment of metagenomic or metatranscriptomic analyses are warranted to establish causal relationships between baicalin, milk microbiota, and fatty acid metabolism. Targeted lipidomics and metabolomics are also required to accurately quantify key metabolites (e.g., glycerophospholipids) closely associated with fatty acid metabolism in dairy cows.

### Conclusion

The present study revealed that dietary supplementation with BAI increased proportions of unsaturated fatty acids in milk through regulating the milk microbiome, milk metabolism, and host metabolism. BAI reshaped the milk microbiome, wherein enriched taxa such as *Myroides* were positively associated with increased milk UFA and altered milk metabolite profiles. Furthermore, BAI regulated some metabolites in milk that were linked to the synthesis of UFA, such as alpha-KG. In addition, BAI regulated host metabolic pathways involved in UFA metabolism, such as primary bile acid biosynthesis. Together, these findings indicated that BAI effectively modifies the milk fatty acid profile to enhance its nutritional quality.

## Data Availability

The data for milk 16S rRNA sequencing and metagenomics are publicly accessible in the NCBI SRA database under accession code PRJNA1332947.

## SUPPLEMENTAL MATERIAL

Supplemental tables and figure spectrum.00836-26-s0001.docx Tables S1 to S4 and Fig. S1.

ASM does not own the copyrights to Supplemental Material that may be linked to, or accessed through, an article. The authors have granted ASM a non-exclusive, world-wide license to publish the Supplemental Material files. Please contact the corresponding author directly for reuse.
