# Supplementary material for: Dietary baicalin supplementation increases the milk unsaturated fatty acid proportion through modulating milk metabolism and the microbiome in dairy cows
Source: Microbiol Spectr. 2026 Oct 7;14(11):e00836-26. doi: 10.1128/spectrum.00836-26 (PMC13729106; doi:10.1128/spectrum.00836-26)
Supplement: Supplementary file 1 [file spectrum.00836-26-s0001.docx]

**Table S1** Chemical composition of the Baicalin (BAI) (% of DM)

| Item | Content |
| --- | --- |
| DM，% as fed | 98.25 |
| OM | 96.37 |
| Total flavonoids | 85.13 |
| Baicalin | 62.78 |
| Chrysin | 9.30 |
| Biochanin A | 2.21 |
| Apigenin | 0.93 |

DM = dry matter; OM = organic matter.

**Table S2** The ingredients and chemical composition of the experimental diets

| Item | Contents |
| --- | --- |
| **Ingredients,% of DM** |  |
| Corn silage | 26.63 |
| Alfalfa hay | 12.77 |
| Ground corn grain | 27.92 |
| Whole cottonseed | 4.42 |
| Molasses skins | 0.72 |
| Oyogold | 0.84 |
| Soybean meal | 13.62 |
| Canolameal | 3.63 |
| Glucose | 1.88 |
| Calcium fatty acid | 2.79 |
| CaCO_3_ | 0.65 |
| NaHCO₃ | 0.52 |
| Nacl | 0.49 |
| Premix^1^ | 3.12 |
| **Chemical composition, % of DM, unless otherwise stated** | |
| DM, % as fed | 48.95 |
| OM | 91.12 |
| CP | 17.73 |
| NDF | 28.59 |
| ADF | 18.64 |
| EE | 4.67 |
| Starch | 27.74 |
| NE_L_ ^2^, Mcal/kg of DM | 1.69 |

DM = dry matter; OM = organic matter; CP = crude protein; NDF = neutral detergent fiber; ADF = acid detergent fiber; EE = ether extract; NE_L_ = Net energy for lactation.

^1^ Formulated to provide (per kg of dry matter): 250,000 IU of vitamin A, 50,000 IU of vitamin D_3_, 1100 IU of vitamin E, 250 mg of Cu, 500 mg of Mn, 1000 mg of Zn, 20 mg of Se, 40 mg of I, 24 mg of Co.

^2^ Net energy for lactation was estimated using the NRC (2001) model.

| **Table S3** Differentially expressed metabolites from the serum between dairy cows that received the CON and BAI | | | | |
| --- | --- | --- | --- | --- |
| Metabolites | VIP | FC | *P*-value | Regulation |
| PC(14:1(9Z)/P-18:1(9Z)) | 1.57 | 0.63 | 0.026 | Down |
| PI(18:0/18:2(9Z,12Z)) | 1.72 | 0.58 | 0.039 | Down |
| Leptomycin B | 3.68 | 0.15 | 0.000 | Down |
| Squamocin_B | 2.39 | 0.50 | 0.021 | Down |
| Butaprost (free acid) | 1.78 | 0.61 | 0.015 | Down |
| Anastrozole | 2.19 | 0.47 | 0.016 | Down |
| Dienestrol diacetate | 2.10 | 0.60 | 0.000 | Down |
| (4OH,8Z,t18:1)_sphingosine | 1.33 | 0.74 | 0.030 | Down |
| 7-Amino-4-methyl-2H-1-benzopyran-2-one arachidonoyl amide | 2.79 | 0.32 | 0.003 | Down |
| rac-trans-4-Cotininecarboxylic acid methyl ester | 2.89 | 0.35 | 0.005 | Down |
| Carnitine | 1.61 | 0.73 | 0.001 | Down |
| 2-Docosahexaenoyl-1-stearoyl-sn-glycero-3-phosphoserine | 1.50 | 0.66 | 0.025 | Down |
| PC(22:6(4Z,7Z,10Z,13Z,16Z,19Z)/22:5(4Z,7Z,10Z,13Z,16Z)) | 1.80 | 0.57 | 0.028 | Down |
| 3-Hydroxy-3H-benzo[de]isochromen-1-one | 1.56 | 0.64 | 0.011 | Down |
| (24R,24(1)R)-Fucosterol epoxide | 2.20 | 0.49 | 0.015 | Down |
| Isoniazid pyruvate | 1.98 | 0.56 | 0.000 | Down |
| Kynurenic acid | 1.18 | 0.74 | 0.032 | Down |
| Ectoine | 2.05 | 0.58 | 0.008 | Down |
| Leucylalanine | 1.88 | 0.59 | 0.049 | Down |
| Hexacosanoic acid | 2.93 | 0.21 | 0.009 | Down |
| Glutarylcarnitine | 1.81 | 0.65 | 0.007 | Down |
| Fagomine | 2.47 | 0.36 | 0.016 | Down |
| Glycolithocholic acid 3-sulfate | 1.91 | 0.54 | 0.032 | Down |
| Glutamate | 1.05 | 0.78 | 0.049 | Down |
| Phenacetin | 1.80 | 0.63 | 0.049 | Down |
| 2-(2,6-Dimethylmorpholin-4-yl)ethanol | 2.34 | 0.54 | 0.000 | Down |
| Ethyl 3-piperidinecarboxylate | 3.34 | 0.18 | 0.008 | Down |
| 3-Piperidin-4-ylpropanoic acid | 1.98 | 0.62 | 0.008 | Down |
| Isocytosine | 1.17 | 0.78 | 0.045 | Down |
| 3-(3-Chlorophenyl)-2,4-imidazolidinedione | 2.93 | 0.30 | 0.000 | Down |
| 1,2,3,4-Tetrahydroisoquinoline-6-carboxylic acid | 1.36 | 0.78 | 0.006 | Down |
| 8-Methylquinolin-2(1H)-one | 2.90 | 0.35 | 0.006 | Down |
| N-Fluorenylacetamide | 2.24 | 0.48 | 0.000 | Down |
| N-Methyl-L-threonine | 2.07 | 0.56 | 0.007 | Down |
| 1-Hexadecyl-2-(8Z,11Z,14Z-eicosatrienoyl)-sn-glycero-3-phosphocholine | 1.49 | 0.75 | 0.005 | Down |
| 1-Behenoyl-2-hydroxy-sn-glycero-3-phosphocholine | 1.50 | 0.68 | 0.027 | Down |
| Isovalerylsarcosine | 1.64 | 0.73 | 0.001 | Down |
| Serotonin | 2.09 | 0.47 | 0.025 | Down |
| 3-Methyl-.gamma.-butyrolactone | 2.06 | 0.61 | 0.003 | Down |
| .DELTA.17-6-Ketoprostaglandin F1.alpha. | 2.47 | 0.43 | 0.030 | Down |
| (3-Ethyl-2-imino-2,3-dihydro-1H-benzimidazol-1-yl)acetic acid | 1.61 | 0.68 | 0.018 | Down |
| 3-(4-Hexyloxyphenyl)succinamic acid | 2.49 | 0.31 | 0.016 | Down |
| **Continued Table S3** Differentially expressed metabolites from the serum between dairy cows that received the CON and BAI | | | | |
| Metabolites | VIP | FC | *P*-value | Regulation |
| Arg-Gly | 2.06 | 0.60 | 0.000 | Down |
| Diethylcarbamazine_N-Oxide | 1.92 | 0.63 | 0.008 | Down |
| 5-[4-(Diethylamino)phenyl]-4-ethyl-4H-1,2,4-triazol-3-yl hydrosulfide | 2.05 | 0.46 | 0.041 | Down |
| Carvedilol | 2.37 | 0.44 | 0.023 | Down |
| Isopropalin | 2.45 | 0.47 | 0.032 | Down |
| (2R)-2-(2,5-Difluorophenyl)pyrrolidine | 1.56 | 0.72 | 0.007 | Down |
| 6-Fluoro-2,3,4,9-tetrahydro-1H-carbazole | 2.86 | 0.36 | 0.004 | Down |
| Deoxycytidine | 1.89 | 0.65 | 0.004 | Down |
| Propanedinitrile, 2-[(3,4,5-trihydroxyphenyl)methylene]- | 1.69 | 0.58 | 0.018 | Down |
| PC(20:3(5Z,8Z,11Z)/P-18:0) | 1.24 | 0.77 | 0.036 | Down |
| PC(14:0/P-16:0) | 1.52 | 0.68 | 0.018 | Down |
| cis-9-Palmitoleic acid | 1.74 | 0.58 | 0.016 | Down |
| (2R)-2-(.beta.-D-Glucopyranosyloxy)-2-phenylacetamide | 4.03 | 12.66 | 0.042 | Up |
| 4-[(2R,3S,7R,8R,8aS)-2,3,4'-Trihydroxy-4,4,7,8a-tetramethyl-6'-oxospiro[2,3,4a,5,6,7-hexahydro-1H-naphthalene-8,2'-3,8-dihydrofuro[2,3-e]isoindole]-7'-yl]butanoic acid | 1.70 | 1.49 | 0.033 | Up |
| 3-(4-Hydroxyphenyl)-3-oxopropyl .beta.-D-glucopyranoside | 1.06 | 1.23 | 0.026 | Up |
| Dimethyl (R)-(+)-malate | 1.70 | 1.76 | 0.034 | Up |
| 3'-O-methylcytidine | 1.11 | 1.21 | 0.017 | Up |
| 7-Ketocholesterol | 1.46 | 1.45 | 0.048 | Up |
| alpha-TOCHOPHERYL ACETATE | 2.27 | 2.16 | 0.020 | Up |
| 2'-O-methylcytidine | 1.11 | 1.21 | 0.017 | Up |
| N-(1H-Tetraazol-5-yl)decanamide | 1.01 | 1.21 | 0.035 | Up |
| L-Arginine, N2-[2-(2,2-diphenylethoxy)acetyl]- | 1.74 | 1.85 | 0.041 | Up |
| CRUSTECDYSONE | 1.96 | 1.97 | 0.022 | Up |
| Ganglioside_GA2_(d18:1/9Z-18:1) | 1.59 | 1.43 | 0.022 | Up |
| Gentamicin | 1.55 | 1.46 | 0.029 | Up |
| 4-Aminobenzoic acid | 1.28 | 1.33 | 0.043 | Up |
| 4-Cyanobenzaldehyde | 1.86 | 1.62 | 0.034 | Up |
| N-Acetylneuraminate | 1.06 | 1.24 | 0.045 | Up |
| 2,6-Dihydroxybenzoic acid | 1.38 | 1.39 | 0.019 | Up |
| Glycohyodeoxycholic acid | 1.75 | 1.85 | 0.027 | Up |
| Dodecanedioic acid | 1.86 | 1.70 | 0.003 | Up |
| Indole-3-propionic acid | 3.22 | 3.70 | 0.001 | Up |
| 2,3-Dihydroxybenzoic acid (Pyrocatechuic acid) | 1.38 | 1.39 | 0.019 | Up |
| Propionylglycine | 1.81 | 1.62 | 0.022 | Up |
| N-Acetylhistidine | 1.34 | 1.30 | 0.017 | Up |
| Methylimidazoleacetic acid | 2.10 | 1.90 | 0.006 | Up |
| Indole-3-pyruvic acid | 1.70 | 1.56 | 0.005 | Up |
| Alfacalcidol | 1.46 | 1.45 | 0.048 | Up |
| Gentisic acid | 1.38 | 1.39 | 0.019 | Up |
| Glycoursodeoxycholic acid | 1.96 | 1.87 | 0.022 | Up |
| **Continued Table S3** Differentially expressed metabolites from the serum between dairy cows that received the CON and BAI | | | | |
| Metabolites | VIP | FC | *P*-value | Regulation |
| Glycodeoxycholic acid | 1.96 | 1.87 | 0.022 | Up |
| 3-Hydroxyvaleric acid | 1.63 | 1.54 | 0.019 | Up |
| Pi-Methylimidazoleacetic acid (hydrochloride) | 2.10 | 1.90 | 0.006 | Up |
| Glycochenodeoxycholic acid | 1.96 | 1.87 | 0.022 | Up |
| Phloroglucinaldehyde | 1.38 | 1.39 | 0.019 | Up |
| 2,4-Dihydroxybenzoic acid | 1.38 | 1.39 | 0.019 | Up |
| Indole-3-methyl acetate | 3.22 | 3.70 | 0.001 | Up |
| 3-Hydroxyisovaleric acid | 1.63 | 1.54 | 0.019 | Up |
| 3-Hydroxyisobutyric acid | 1.45 | 1.55 | 0.042 | Up |
| Paeonol | 1.98 | 1.66 | 0.016 | Up |
| N-(1H-Indol-3-ylacetyl)glycine | 1.75 | 1.51 | 0.000 | Up |
| 1-Docosahexaenoyl-2-stearoyl-sn-glycero-3-phosphocholine | 1.06 | 1.21 | 0.041 | Up |
| 4-Amino-6-methoxy-3-quinolinecarboxylic acid | 1.72 | 1.61 | 0.024 | Up |
| Quinolin-6-yl-acetic acid, methyl ester | 5.68 | 9.58 | 0.018 | Up |
| 2-Fluoro-5-nitrophenyl alcohol | 1.79 | 1.60 | 0.024 | Up |
| Hexadecanedioic acid, 3,3,14,14-tetramethyl- | 2.13 | 2.03 | 0.012 | Up |
| 2-Hydroxy-6-methylquinoline-3-carbaldehyde | 1.20 | 1.29 | 0.046 | Up |
| PC(37:3) | 1.58 | 1.43 | 0.012 | Up |
| Chenodeoxycholic acid 24-acyl-.beta.-D-glucuronide | 2.52 | 3.51 | 0.012 | Up |
| 3,5,9-Trioxa-4-phosphatetracosan-1-aminium, 7-(acetyloxy)-24-carboxy-4-hydroxy-N,N,N-trimethyl-, inner salt, 4-oxide, (R)- | 1.80 | 1.57 | 0.001 | Up |
| 5-Aminopentanal | 2.82 | 3.03 | 0.007 | Up |
| Trimethylamine N-oxide | 1.24 | 1.28 | 0.034 | Up |
| L-.beta.-Homomethionine | 1.57 | 1.45 | 0.048 | Up |
| LPE(18:1(9Z)/0:0) | 1.65 | 1.49 | 0.009 | Up |
| 2,4-Dichloro-5-ethyl-3-methylphenol | 1.57 | 1.44 | 0.000 | Up |
| PC(P-16:0/0:0) | 1.44 | 1.28 | 0.001 | Up |
| Tyramine_glucuronide | 3.46 | 6.17 | 0.013 | Up |
| 13,14-Dihydro-16,16-difluoroprostaglandin J2 | 1.45 | 1.45 | 0.046 | Up |
| Atrazine-desethyl-desisopropyl | 2.54 | 2.29 | 0.003 | Up |
| 2-(2-Chloroethyl)-6-fluoro-1H-benzimidazole | 1.98 | 1.73 | 0.005 | Up |
| 3-Amino-5-sulfosalicyclicacid | 1.45 | 1.48 | 0.023 | Up |
| 7C-aglycone | 1.79 | 1.62 | 0.025 | Up |
| 3-Hydroxybutyric acid | 1.45 | 1.55 | 0.042 | Up |
| N-Acetylmethionine | 1.51 | 1.48 | 0.030 | Up |
| Cytochalasin_Npho | 1.36 | 1.37 | 0.039 | Up |
| Hymeglusin | 1.87 | 1.92 | 0.007 | Up |
| (S)-3,5-Dihydroxyphenylglycine | 1.39 | 1.33 | 0.001 | Up |
| PC(20:3(5Z,8Z,11Z)/15:0) | 1.65 | 1.44 | 0.002 | Up |
| Bonactin | 1.46 | 1.57 | 0.048 | Up |
| PC(16:1(9Z)/0:0)[U] | 1.30 | 1.29 | 0.012 | Up |

VIP = ariable importance in projection; FC = fold change; CON = control diet with no Baicalin (BAI) supplementation (n = 6); BAI = control diet with 25 g/d BAI supplementation per cow (n = 6).

| **Table S4** Differentially expressed metabolites from the milk between dairy cows that received the CON and BAI | | | | |
| --- | --- | --- | --- | --- |
| Metabolites | VIP | FC | *P*-value | Regulation |
| sn-Glycerol 3-phosphate | 1.36 | 0.54 | 0.014 | Down |
| Hematoxylin | 1.45 | 0.55 | 0.003 | Down |
| 1,1'-Binaphthyl-8,8'-dicarboxylic acid | 2.36 | 0.25 | 0.048 | Down |
| 2,6-Bis(2-benzimidazolyl)pyridine | 2.01 | 0.14 | 0.038 | Down |
| 6-Tuliposide A | 1.48 | 0.51 | 0.004 | Down |
| Uracil 5-carbaldehyde | 2.34 | 0.30 | 0.000 | Down |
| 1-Deoxy-D-altro-heptulose 7-phosphate | 1.72 | 0.42 | 0.001 | Down |
| sn-Glycerol 1-phosphate | 1.26 | 0.60 | 0.016 | Down |
| Ureidosuccinic acid | 1.17 | 0.61 | 0.033 | Down |
| Aspartate | 1.44 | 0.58 | 0.001 | Down |
| N-Acetylmannosamine | 1.25 | 0.56 | 0.036 | Down |
| Phosphoglycolic acid | 1.70 | 0.45 | 0.004 | Down |
| Pyrophosphate | 1.34 | 0.57 | 0.021 | Down |
| 6,8-Dichloro-4-quinazolinol | 1.29 | 0.56 | 0.028 | Down |
| (S)-3,4-Dihydroxybutyric acid (lithium hydrate) | 1.13 | 0.63 | 0.041 | Down |
| 2-Fucosyllactose | 2.08 | 0.23 | 0.019 | Down |
| 6'-Sialyl-N-acetyllactosamine | 1.86 | 0.42 | 0.003 | Down |
| {[3-Ethoxy-2-(ethoxycarbonyl)-3-oxo-1-propenyl]amino}acetic acid | 1.38 | 0.56 | 0.017 | Down |
| 3'-N-Acetylneuraminyl-N-acetyllactosamine | 1.75 | 0.40 | 0.009 | Down |
| 3-Phosphonopropanoic acid | 1.88 | 0.37 | 0.002 | Down |
| Lumichrome | 1.27 | 0.64 | 0.004 | Down |
| Permalon Orange II | 1.12 | 0.65 | 0.028 | Down |
| Avenic_acid_B | 1.57 | 0.41 | 0.028 | Down |
| 2'-Chloro-2-hydroxy-5-methylbenzophenone | 1.03 | 0.67 | 0.045 | Down |
| 1-[4-(4-Quinazolinylamino)phenyl]ethanone | 1.20 | 0.62 | 0.032 | Down |
| 7-Chloro-5-(1H-pyrrol-2-yl)-1,3-dihydro-1,4-benzodiazepin-2-one | 1.41 | 0.54 | 0.017 | Down |
| 6-Ethoxy-4-methylcoumarin | 1.66 | 0.40 | 0.018 | Down |
| SB 202190 | 1.32 | 0.55 | 0.022 | Down |
| 3-[N,N-Bis(2-hydroxyethyl)amino]-2-hydroxypropanesulfonic acid | 1.13 | 0.64 | 0.028 | Down |
| Piceatannol | 1.11 | 0.65 | 0.030 | Down |
| Dodecanoic acid | 1.58 | 0.49 | 0.013 | Down |
| Capric acid | 1.80 | 0.34 | 0.021 | Down |
| Caprylic acid | 1.83 | 0.31 | 0.028 | Down |
| Stearic acid | 1.90 | 0.37 | 0.006 | Down |
| Phosphoenolpyruvic acid | 2.39 | 2.49 | 0.040 | Up |
| cis-11-Eicosenoic acid | 2.08 | 3.63 | 0.007 | Up |
| Cohibin_D | 1.40 | 1.73 | 0.037 | Up |
| PS(20:3(8Z,11Z,14Z)/18:2(9Z,12Z)) | 1.11 | 1.42 | 0.037 | Up |
| (2-aminoethoxy)[2-[docosa-4.7.10.13.16.19-hexaenoyloxy]-3-[octadeca-1.9-dien-1-yloxy]propoxy]phosphinic acid | 2.14 | 2.96 | 0.011 | Up |
| 2-Hydroxystearic acid | 1.73 | 2.31 | 0.039 | Up |
| PS(18:2(9Z,12Z)/16:0) | 1.57 | 1.88 | 0.010 | Up |
| **Continued Table S4** Differentially expressed metabolites from the milk between dairy cows that received the CON and BAI | | | | |
| Metabolites | VIP | FC | *P*-value | Regulation |
| PS(18:0/20:3(8Z,11Z,14Z)) | 1.79 | 2.12 | 0.018 | Up |
| PS(16:0/16:1(9Z)) | 1.95 | 2.44 | 0.019 | Up |
| Simvastatin 2M+NH4 | 1.28 | 1.61 | 0.043 | Up |
| PC(20:0/22:6(4Z,7Z,10Z,13Z,16Z,19Z)) | 1.66 | 2.02 | 0.026 | Up |
| PE(P-16:0/18:1(9Z)) | 1.20 | 1.58 | 0.031 | Up |
| 3,4-Methylenedioxybenzyl methyl ketoximine | 1.65 | 2.05 | 0.003 | Up |
| LysoPE(0:0/18:0) | 1.46 | 1.76 | 0.049 | Up |
| (2-aminoethoxy)[2-[icosa-5.8.11-trienoyloxy]-3-(octadecanoyloxy)propoxy]phosphinic acid | 1.28 | 1.60 | 0.032 | Up |
| 4-(3,5-Dichloroanilino)-4-oxobutanoic acid | 1.49 | 1.85 | 0.010 | Up |
| PE(22:4(7Z,10Z,13Z,16Z)/22:2(13Z,16Z)) | 2.33 | 3.25 | 0.003 | Up |
| 4-(Methylmercapto)phenol | 1.37 | 1.70 | 0.035 | Up |
| 5-Taurinomethyl-2-thiouridine | 2.06 | 2.54 | 0.030 | Up |
| Cefdinir | 1.25 | 1.71 | 0.048 | Up |
| 16,16-Dimethyl-6-ketoprostaglandin E1 | 2.26 | 4.15 | 0.021 | Up |
| (5.Xi.,9.Xi.,18.Xi.)-3-Oxolup-20(29)-ene-27,28-dioic acid | 2.49 | 5.05 | 0.039 | Up |
| Crustecdysone | 1.75 | 2.39 | 0.016 | Up |
| Glycine, N,N'-[1,2-ethanediylbis(oxy-2,1-phenylene)]bis[N-(carboxymethyl)- | 1.48 | 1.84 | 0.007 | Up |
| 1H-Imidazole-1-acetic_acid | 1.40 | 1.66 | 0.026 | Up |
| N-Benzoyl-4-methylbenzenesulfonamide | 1.34 | 1.58 | 0.017 | Up |
| N-(4-Nitrobenzoyl)phenylalanine | 2.40 | 3.90 | 0.003 | Up |
| PC(20:1(11Z)/22:4(7Z,10Z,13Z,16Z)) | 1.56 | 1.87 | 0.037 | Up |
| 5-(chloromethyl)-1H-tetraazole | 2.14 | 2.82 | 0.001 | Up |
| 2-Aminoadipic acid | 1.02 | 1.41 | 0.019 | Up |
| S-(Indolylmethylthiohydroximoyl)-L-cysteine | 1.56 | 2.05 | 0.006 | Up |
| IDP | 2.58 | 2.28 | 0.043 | Up |
| poxystearic acid | 1.72 | 2.72 | 0.040 | Up |
| Linoleate | 1.35 | 1.81 | 0.035 | Up |
| L-Alanyl-gamma-D-glutamyl-L-lysine | 1.43 | 1.69 | 0.029 | Up |
| omega-Muricholic acid | 2.46 | 4.83 | 0.049 | Up |
| Isovalerylglycine | 2.00 | 2.61 | 0.001 | Up |
| Indoxyl sulfate | 1.57 | 1.95 | 0.003 | Up |
| Glucose 1-phosphate | 1.54 | 1.84 | 0.045 | Up |
| 9-Oxooctadecanoic acid | 1.72 | 2.72 | 0.040 | Up |
| 2-Methylbutyrylglycine | 2.00 | 2.61 | 0.001 | Up |
| Butyrylcarnitine (Car(4:0)) | 1.86 | 2.08 | 0.000 | Up |
| Valerylcarnitine | 1.43 | 1.70 | 0.004 | Up |
| 2-Hydroxyoctanoic acid | 1.69 | 3.10 | 0.050 | Up |
| 16-Hydroxypalmitic acid | 1.69 | 3.10 | 0.045 | Up |
| 2-Hydroxypalmitic acid | 1.69 | 3.10 | 0.045 | Up |
| LPC(13:0) | 1.61 | 1.95 | 0.017 | Up |
| **Continued Table S4** Differentially expressed metabolites from the milk between dairy cows that received the CON and BAI | | | | |
| Metabolites | VIP | FC | *P*-value | Regulation |
| Isobutyrylcarnitine (Car(4:0)) | 1.86 | 2.08 | 0.000 | Up |
| Phenylacetylglycine | 1.82 | 2.25 | 0.001 | Up |
| 2-Methylhippuric acid | 1.82 | 2.25 | 0.001 | Up |
| LPE(14:0) | 1.77 | 2.20 | 0.032 | Up |
| Galactose 1-phosphate | 1.47 | 1.78 | 0.035 | Up |
| LPC(18:2/0:0) | 1.70 | 2.17 | 0.019 | Up |
| Mannose 1-phosphate | 1.47 | 1.78 | 0.035 | Up |
| Isovalerylcarnitine (Car(5:0)) | 1.43 | 1.70 | 0.004 | Up |
| 12-Hydroxystearic acid | 1.73 | 2.31 | 0.039 | Up |
| Prolylhydroxyproline | 1.97 | 2.68 | 0.002 | Up |
| Sphingosine | 1.95 | 2.45 | 0.010 | Up |
| 2-methylcitrate | 1.38 | 1.68 | 0.022 | Up |
| Hyocholic acid | 2.46 | 4.83 | 0.049 | Up |
| 3-Hydroxyoctanoic acid | 1.69 | 3.10 | 0.050 | Up |
| Citraconic acid | 1.32 | 1.53 | 0.001 | Up |
| alpha-Muricholic acid | 2.46 | 4.83 | 0.049 | Up |
| Pehanorm | 1.26 | 1.67 | 0.048 | Up |
| 5-Hydroxyvalproic acid | 1.69 | 3.10 | 0.050 | Up |
| 4-Hydroxyproline | 1.47 | 2.03 | 0.011 | Up |
| beta-Muricholic acid | 2.46 | 4.83 | 0.049 | Up |
| 5-Aminolevulinic acid | 1.47 | 2.03 | 0.011 | Up |
| PC(14:0/16:0) | 1.27 | 1.57 | 0.011 | Up |
| 5,2'-O-Dimethylcytidine | 1.54 | 1.81 | 0.005 | Up |
| Trimethylamine N-oxide | 1.51 | 1.72 | 0.008 | Up |
| Fagomine | 2.07 | 2.54 | 0.017 | Up |
| Threonic acid | 1.20 | 1.49 | 0.003 | Up |
| 3-Hydroxyhippuric acid | 1.41 | 1.87 | 0.036 | Up |
| 7-Hydroxy-4-(trifluoromethyl)coumarin | 1.79 | 2.17 | 0.025 | Up |
| 1-Aminopropan-2-ol | 1.78 | 1.92 | 0.000 | Up |
| HEPTANOIC ACID | 1.43 | 1.82 | 0.031 | Up |
| Glycerophospho-N-palmitoylethanolamine | 1.76 | 2.15 | 0.006 | Up |
| Pseudouridine | 1.07 | 1.38 | 0.015 | Up |
| (12Z)-9,10,11-Trihydroxyoctadec-12-enoic acid | 2.15 | 4.03 | 0.021 | Up |
| 10-Methylundecanoic acid | 1.89 | 3.30 | 0.018 | Up |
| PC(31:1) | 1.27 | 1.57 | 0.012 | Up |
| Ursocholic acid | 2.46 | 4.83 | 0.049 | Up |
| Piperidine | 1.75 | 2.05 | 0.001 | Up |
| cis-5-Dodecenoic acid | 1.00 | 1.39 | 0.031 | Up |
| Resorcinol | 1.32 | 1.64 | 0.018 | Up |
| Glycerophospho-N-oleoylethanolamine | 1.62 | 2.22 | 0.026 | Up |
| 2-Oxiraneoctanoic acid, .eta.-hydroxy-3-octyl- | 2.27 | 4.74 | 0.009 | Up |
| PC(35:4) | 1.32 | 1.56 | 0.010 | Up |
| **Continued Table S4** Differentially expressed metabolites from the milk between dairy cows that received the CON and BAI | | | | |
| Metabolites | VIP | FC | *P*-value | Regulation |
| Isocaproic acid | 1.96 | 3.78 | 0.028 | Up |
| 12(13)-Epoxy-9Z-octadecenoic acid | 1.63 | 2.94 | 0.049 | Up |
| (6E)-8-Methyl-6-nonenoic acid | 1.97 | 3.23 | 0.012 | Up |
| 1,2-Dioleoyl-sn-glycero-3-phosphoethanolamine-N-methyl | 1.45 | 1.80 | 0.046 | Up |
| N-(1H-Indol-3-ylacetyl)glycine | 2.13 | 3.14 | 0.002 | Up |
| Allocholic acid | 2.46 | 4.83 | 0.049 | Up |
| 5-(2-Furyl)-4H-1,2,4-triazol-3-amine | 1.11 | 1.50 | 0.028 | Up |
| Cholic acid | 2.46 | 4.83 | 0.049 | Up |
| PI(16:0/18:1) | 1.46 | 1.77 | 0.044 | Up |
| [2-[docosa-7.10.13.16-tetraenoyloxy]-3-(octadecanoyloxy)propoxy]({[2.3.4.5.6-pentahydroxycyclohexyl]oxy})phosphinic acid | 1.90 | 2.29 | 0.039 | Up |
| 3-(2-Aminoethylthio)-2-methylpropionic acid | 1.57 | 1.78 | 0.003 | Up |
| 2-[(2-Aminoethyl)sulfanyl]acetic acid | 1.97 | 2.42 | 0.000 | Up |
| 2-Hydroxyglutaric acid | 1.83 | 3.10 | 0.009 | Up |
| 2-Fluoro-5-nitrobenzenemethanol | 1.29 | 1.51 | 0.039 | Up |
| 1-(2-Hydroxyethyl)pyrazole | 2.58 | 4.46 | 0.001 | Up |
| PC(35:3) | 1.41 | 1.86 | 0.025 | Up |
| PE(P-36:4) | 1.80 | 2.15 | 0.002 | Up |
| Propionylglycine | 1.47 | 2.03 | 0.011 | Up |
| 2-Oleoyl-1-palmitoyl-sn-glycero-3-phosphoserine | 1.56 | 1.92 | 0.047 | Up |
| LPE(18:1(9Z)/0:0) | 1.65 | 2.31 | 0.023 | Up |
| 1,2-Dioleoyl-sn-glycero-3-phosphoethanolamine-N,N-dimethyl | 1.16 | 1.51 | 0.049 | Up |
| Ethyl 6-chloro-4-hydroxyquinoline-3-carboxylate | 1.58 | 2.02 | 0.025 | Up |
| 1-Mercapto[1,2,4]triazolo[4,3-a]quinoxalin-4(5H)-one | 1.35 | 1.67 | 0.031 | Up |
| 2,4-Dichloro-5-ethyl-3-methylphenol | 1.36 | 1.72 | 0.002 | Up |
| 1-Stearoyl-2-arachidonoyl-sn-glycero-3-phosphoserine | 1.64 | 1.97 | 0.032 | Up |
| Valproic acid | 1.83 | 3.18 | 0.028 | Up |
| 1-Palmitoyl-2-arachidonoyl-sn-glycero-3-phosphoserine | 1.50 | 2.02 | 0.020 | Up |
| 2-Arachidonoyl-1-palmitoyl-sn-glycero-3-phosphoethanolamine | 1.21 | 1.58 | 0.034 | Up |
| cis-11.14-Eicosadienoic acid | 2.43 | 4.68 | 0.001 | Up |
| 1-Stearoyl-2-arachidonoyl-sn-glycero-3-phospho-(1'-myo-inositol) | 1.91 | 2.51 | 0.007 | Up |
| Galabiosylceramide_(d18:1/20:0) | 1.82 | 2.04 | 0.004 | Up |
| 5-(4-Methoxyphenyl)-7-(trifluoromethyl)pyrazolo[1,5-a]pyrimidine-2-carboxylic acid | 1.42 | 1.70 | 0.007 | Up |
| Ikarugamycin | 1.50 | 2.25 | 0.035 | Up |
| 2-Arachidonoyl-1-stearoyl-sn-glycero-3-phosphoethanolamine | 1.33 | 1.71 | 0.028 | Up |
| PC(38:7) | 1.45 | 1.71 | 0.018 | Up |
| Caproic acid | 1.96 | 3.78 | 0.028 | Up |
| alpha-Ketoglutaric acid (alpha-KG) | 1.18 | 1.50 | 0.032 | Up |
| 2-Hydroxy-2-[2-[(Z)-2-hydroxy-2,6-dimethyl-8-(7-oxofuro[3,2-g]chromen-4-yl)oxyoct-6-en-3-yl]oxy-2-oxoethyl]butanedioic acid | 1.60 | 2.21 | 0.010 | Up |
| **Continued Table S4** Differentially expressed metabolites from the milk between dairy cows that received the CON and BAI | | | | |
| Metabolites | VIP | FC | *P*-value | Regulation |
| Tetrafluorosuccinic acid | 1.31 | 1.54 | 0.001 | Up |
| acetohydroxamic_acid | 1.77 | 2.18 | 0.001 | Up |
| 2-amino-3-({hydroxy[2-[icosa-5.8.11-trienoyloxy]-3-(octadecanoyloxy)propoxy]phosphoryl}oxy)propanoic acid | 1.70 | 2.07 | 0.039 | Up |
| (2-aminoethoxy)[3-[hexadec-1-en-1-yloxy]-2-[icosa-5.8.11.14-tetraenoyloxy]propoxy]phosphinic acid | 1.82 | 2.21 | 0.009 | Up |
| PI(20:3(5Z,8Z,11Z)/18:1(9Z)) | 1.58 | 1.81 | 0.001 | Up |
| 2-Ketobutyric acid | 1.21 | 1.65 | 0.035 | Up |
| Pentadecanoic acid | 2.08 | 3.04 | 0.002 | Up |
| (2-aminoethoxy)[2-[hexadec-9-enoyloxy]-3-[octadeca-1.11-dien-1-yloxy]propoxy]phosphinic acid | 1.34 | 1.64 | 0.038 | Up |
| PC(20:3(5Z,8Z,11Z)/15:0) | 1.19 | 1.45 | 0.012 | Up |
| PC(15:0/16:0) | 1.30 | 1.61 | 0.047 | Up |
| Syringetin 3-galactoside | 1.54 | 1.87 | 0.023 | Up |
| (2-aminoethoxy)[3-[hexadec-1-en-1-yloxy]-2-[hexadec-9-enoyloxy]propoxy]phosphinic acid | 1.38 | 1.67 | 0.027 | Up |
| PS(22:6(4Z,7Z,10Z,13Z,16Z,19Z)/16:0) | 1.28 | 1.56 | 0.008 | Up |
| PC(20:3(5Z,8Z,11Z)/14:0) | 1.17 | 1.51 | 0.032 | Up |
| PE(18:2(9Z,12Z)/0:0) | 1.60 | 2.36 | 0.025 | Up |
| N-(2,5-Dichlorophenyl)-2-hydroxybenzamide | 1.00 | 1.28 | 0.040 | Up |

VIP = ariable importance in projection; FC = fold change; CON = control diet with no Baicalin (BAI) supplementation (n = 6); BAI = control diet with 25 g/d BAI supplementation per cow (n = 6).

**Figure S1** (A-B) PCA plot of serum metabolome in positive mode (A) and negative mode (B). (C-D) PCA plot of milk metabolome in positive mode (C) and negative mode (D). CON = control diet with no Baicalin (BAI) supplementation (n = 6); BAI = control diet with 25 g/d BAI supplementation per cow (n = 6).
